# Selective Blockade of HCN1/HCN2 Channels as a Potential Pharmacological Strategy Against Pain

**DOI:** 10.3389/fphar.2018.01252

**Published:** 2018-11-08

**Authors:** Leonardo Dini, Martina Del Lungo, Francesco Resta, Michele Melchiorre, Valentina Spinelli, Lorenzo Di Cesare Mannelli, Carla Ghelardini, Annunziatina Laurino, Laura Sartiani, Raffaele Coppini, Guido Mannaioni, Elisabetta Cerbai, Maria Novella Romanelli

**Affiliations:** Department of Neurosciences, Psychology, Drug Research and Child Health (NeuroFarBa), University of Florence, Florence, Italy

**Keywords:** neuropathic pain, dorsal root ganglion neurons, hyperpolarization-activated current, HCN channel blockade, oxaliplatin

## Abstract

A prominent role of hyperpolarization-activated, cyclic nucleotide-gated (HCN) channels has been suggested based on their expression and (dys)function in dorsal root ganglion (DRG) neurons, being likely involved in peripheral nociception. Using HCN blockers as antinociceptive drugs is prevented by the widespread distribution of these channels. However, tissue-specific expression of HCN isoforms varies significantly, HCN1 and HCN2 being considered as major players in DRG excitability. We characterized the pharmacological effect of a novel compound, MEL55A, able to block selectively HCN1/HCN2 isoforms, on DRG neuron excitability *in-vitro* and for its antiallodynic properties *in-vivo*. HEK293 cells expressing HCN1, HCN2, or HCN4 isoforms were used to verify drug selectivity. The pharmacological profile of MEL55A was tested on mouse DRG neurons by patch-clamp recordings, and *in-vivo* in oxaliplatin-induced neuropathy by means of thermal hypersensitivity. Results were compared to the non-isoform-selective drug, ivabradine. MEL55A showed a marked preference toward HCN1 and HCN2 isoforms expressed in HEK293, with respect to HCN4. In cultured DRG, MEL55A reduced *I*_h_ amplitude, both in basic conditions and after stimulation by forskolin, and cell excitability, its effect being quantitatively similar to that observed with ivabradine. MEL55A was able to relieve chemotherapy-induced neuropathic pain. In conclusion, selective blockade of HCN1/HCN2 channels, over HCN4 isoform, was able to modulate electrophysiological properties of DRG neurons similarly to that reported for classical *I*_h_ blockers, ivabradine, resulting in a pain-relieving activity. The availability of small molecules with selectivity toward HCN channel isoforms involved in nociception might represent a safe and effective strategy against chronic pain.

## Introduction

During the last decade, hyperpolarizing activated cyclic nucleotide-gated (HCN) channels emerged as key players controlling and facilitating neuron excitability. The Na^+^/K^+^ inward current flowing during HCN opening, *I*_h_, appears to contribute to spontaneous or ectopic firing in several tissues, including central nervous system and peripheral ganglia and nerves (recently reviewed in Sartiani et al., 2017). Among the most interesting ones, because of potential pathophysiological implications, are the nociceptive neurons whose bodies reside in the dorsal root ganglia (DRG). Recent promising findings demonstrate that *I*_h_ activation plays a facilitating role in neuropathic pain, an ill-treated disease demanding new pharmacological strategies (Tsantoulas et al., 2016). Several pieces of evidence support the over-expression and/or gain of function of HCN in animal models of chronic, neuropathic pain (Chaplan et al., 2003; Yao et al., 2003; Jiang et al., 2008). Mechanisms triggering hyperalgesia or allodynia may involve gene reprogramming (Papp et al., 2010; Descoeur et al., 2011; Schnorr et al., 2014) as well as cAMP-mediated channel gating consequent to receptor stimulation by prostaglandin E2 and substance P (Jafri and Weinreich, 1998; Momin et al., 2008; Resta et al., 2016).

A major limitation in assessing and exploiting the pharmacological impact of HCN modulation in pain nociception is the lack of isoform-selective compounds. The HCN family consists of four main isoforms (HCN1-4), assembling as homo- or heterotetramers in the naïve channels, whose biophysical properties and tissue distribution differ substantially within the nervous system and beyond (Biel et al., 2009). As a matter of fact, ivabradine, the only clinically available HCN blocker, exerts a specific, bradycardic action entailed in chronic angina and heart failure, yet unwarranted in neurological disorders (Savalieva and Camm, 2006). Ivabradine is a not-isoform selective drug and the heart rate-reducing effect has been attributed mainly to HCN4 blockade, the most highly expressed isoform in the sinoatrial node (Sartiani et al., 2017). DRG neurons express HCN isoforms unevenly, with HCN1 predominating in large neurons, HCN2 in small-medium ones and HCN3-4 scarcely present in all of them (Acosta et al., 2012; Schnorr et al., 2014). Altogether, these data suggest that isoform-selective HCN blockers might represent novel and safe analgesic arms against neuropathic pain (Tibbs et al., 2016; Tsantoulas et al., 2016).

The feasibility of an antinociceptive strategy based on isoform-selective HCN blockers has been confirmed by a recent paper by some of us in a rat model of neuropathic pain (Resta et al., 2018). These results were obtained using MEL57A, a phenylalkylamine structurally related to zatebradine, displaying significant selectivity for HCN1 over HCN2 and HCN4 (Melchiorre et al., 2010). Indeed, previous structure-activity studies from our lab suggested that naïve *I*_h_ current, recorded in DGR neurons, was preferentially reduced by this HCN1-selective blocker, when compared to another derivative (EC18) showing selectivity for HCN4 (Del Lungo et al., 2012). However, due to the relevant function of HCN2 isoform in the transmission of painful stimuli (Emery et al., 2011) and the possibility that HCN1/HCN2 co-assemble in heterotetramers (Chen et al., 2001), a strategy based on HCN1/HCN2 blockade (but not HCN4 blockade) might represent a promising option.

Based on preliminary proof of concept, we focused our attention on MEL55A, previously synthesized by us, showing an interesting pharmacological profile, being able to preferentially block HCN1 and HCN2 over HCN4 (Melchiorre et al., 2010). MEL55A is a reduced-flexibility analog of zatebradine, differing from the lead by the presence of a *cis*-butene moiety in place of the three-methylene chain, an endocyclic double bond, and a stereogenic center (R configuration) close to the dimethoxyphenyl ring (Supplemental Figure 1). In the present work, we aimed to test whether the potency of this compound translates into a modulatory effect of DRG neuron excitability and underlying *I*_h_, and into an antihyperalgesic activity. These effects were compared with those of the non-selective drug, ivabradine.

## Materials and methods

### HEK culture

Human embryonic kidney cells (HEK293 cells DSMZ, Braunschweig, Germany), transfected with mouse HCN1 (mHCN1), mouse HCN2 (mHCN2), and human HCN4 (hHCN4) cDNA (provided by Prof. M. Biel, Ludwig-Maximilians-Universität München), were cultured as described previously (Del Lungo et al., 2012) in DMEM medium (DMEM + GlutaMaxTM-I x1, Gibco, Italy) supplemented with 10% fetal bovine serum (FBS), 100 U/ml penicillin, 100 μg/ml streptomycin and 200 μg/ml geneticin (G418, Gibco, Italy) in T25 flasks and incubated at 37°C with 5% CO_2_. At confluence (3–5 days after plating), cells were detached by using trypsin-EDTA and the sedimented cells were either re-plated or used for electrophysiological measurements. Prior to electrophysiological recordings, HEK293 cells were incubated in Tyrode's solution (see Solutions) in the presence of 300 μM CaCl_2_ for 2–3 h at room temperature.

### Animals

Behavioral tests were performed on male CD-1 albino mice (Envigo, Italy) weighing ~22–25 g at the beginning of the experimental procedure, were used. *In vitro* measurements were performed on dorsal root ganglia (DRG) of C57black mice, 3–8 months (Envigo, Italy). Animals were housed in CeSAL (Centro Stabulazione Animali da Laboratorio, University of Florence) and used at least 1 week after their arrival. Ten mice were housed per cage (size 26 × 41 cm); animals were fed a standard laboratory diet and tap water *ad libitum*, and kept at 23 ± 1°C with a 12 h light/dark cycle, light at 7 a.m. The experimental protocol was carried out after approval by the Animal Care and Research Ethics Committee of the University of Florence, Italy, under license from the Italian Department of Health and in compliance with the Directive 2010/63/EU of the European parliament and of the European Union council (22 September 2010) on the protection of animals used for scientific purposes. The ethical policy of the University of Florence complies with the Guide for the Care and Use of Laboratory Animals of the US National Institutes of Health (NIH Publication No. 85-23, revised 1996; University of Florence assurance number: A5278-01). Experiments involving animals have been reported according to ARRIVE guidelines [McGrath, 2015 #485]. All efforts were made to minimize animal suffering and to reduce the number of animals used.

### Mouse dorsal root ganglion neurons preparation

Experiments were performed on dorsal root ganglia (DRG) of adult mice (C57black, 6–8 weeks). A total number of 12 mice have been used for this study. Twenty-thirty ganglia were isolated from the full length of the spinal column following removal of the spinal cord and used for primary cultures or Western blots.

For primary cultures of DRG neurons, after incubation in collagenase (2.5 mg/ml) for 1 h at 37°C, ganglia were mechanically triturated with a 45 μm sterile needle. The cell suspension was filtered in 40 μm Nylon filter (BD Falcon) then centrifuged and re-suspended in Dulbecco's modified Eagle's medium (DMEM, Gibco, Italy) supplemented with 50 U/ml penicillin and 0.05 mg/ml streptomycin (Invitrogen), 1% L-glutamine (Invitrogen), 10% fetal bovine serum (FBS, Gibco, Italy), 50 ng/ml nerve growth factor (NGF, Promega) and 1.25 μg/ml cytosine β-D-arabinofuranoside (Ara-C, Sigma, Italy). DRG neurons were plated onto 13 mm borosilicate cover glass previously coated with polyL-lysine (100 μg/ml, Sigma, Italy) and laminin (10 μg/ml, Sigma, Italy). The medium was changed after 24 h. Immunocytochemistry staining and electrophysiological recordings were made within 60–72 h of culture; during this time, cells develop in most cases neurites as previously reported (Fukuda, 1985).

### Oxaliplatin-induced neuropathy

Mice treated with oxaliplatin (2.4 mg/kg) were administered i.p. on days 1–2, 5–9, 12–14 (10 i.p. injections) (Cavaletti et al., 2001; Di Cesare Mannelli et al., 2017). Oxaliplatin was dissolved in 5% glucose solution. Control animals received an equivalent volume of vehicle. Behavioral tests were performed on day 15.

### Cold plate test

The animals were placed in a stainless-steel box (12 × 20 × 10 cm) with a cold plate as floor. The temperature of the cold plate was kept constant at 4 ± 1°C. Pain-related behavior (licking of the hind paw) was observed and the time (seconds) of the first sign was recorded. The cut-off time of the latency of paw lifting or licking was set at 60 s (Di Cesare Mannelli et al., 2013).

### Immunocytochemistry

DRG neurons were fixed in 4% paraformaldehyde in PBS for 15 min and permeabilized in 0.3% Triton X-100-PBS for 10 min. Cells were then blocked in 1% BSA for 10 min and incubated with rabbit anti-HCN1 [1:300], rabbit anti-HCN2 [1:100], rabbit anti-HCN3 [1:25], rabbit anti-HCN4 [1:200] (Alomone Labs, Israel) antibodies overnight at 4°C and Alexa Fluor 546 anti-rabbit (Invitrogen) secondary antibodies for 2 h. To spot nuclei, the sample was incubated with, 4′, 6-diamidino-2-phenylindole [1:1000] (DAPI, Vectashield Labs, UK) in 0.1% Tween 20 in PBS for 10 min. Images were obtained using a fluorescence microscope (Olympus BX63, Italy) with a 20X objective and a CellSens Dimension Imaging Software (Olympus, Italy). HCN immunofluorescence in cultured DRG neurons was semi-quantitatively measured on a computer using ImageJ 1.33 image analysis software (http://rsb.info.nih.gov/ij), as described in Bigagli et al. (2018). Briefly, eight photomicrographs were randomly taken of each sample and for each cell total, membrane, or cytoplasmic HCN fluorescence was measured and expressed as pixels. These values were used to calculate the membrane/cytoplasmic relative fluorescence (% total cell fluorescence) of HCN channels.

### Patch-clamp experiments

Single cell patch-clamp experiments were performed in the whole-cell configuration using a PC-505B amplifier (Warner, Handen, CT, USA) and digitalized with Digidata 1440 A and Clampex 10 (Axon, Sunnyvale, CA,USA). Pipettes, resistance 3–5 MΩ, were pulled from borosilicate capillaries (Harvard Apparatus Ltd, Kent, U.K.) using a two-stage horizontal puller (model P-87; Sutter Instrument, Novato, CA). Signals were sampled at 10 kHz and low-pass filtered at 1 kHz. All recordings were made at room temperature. Cells were continuously perfused with extracellular solution using a gravity-fed perfusion system. We patched cells with a diameter <30 μm thus including the large majority of nociceptive neuron. Membrane capacitance (C_m_) was measured by applying a ±10 mV pulse from a holding potential of −40 mV. Only cells showing stable C_m_ and series resistance (R_s_) were included in the analysis.

Action potential (AP) recordings were performed in DRG neurons using a protocol constituted by a hyperpolarizing current step (−100 pA, duration: 1 s) followed by a series of depolarizing steps of increasing intensity (from 20 to 100 pA, duration: 1 s), to evoke the voltage-sag and the AP, respectively.

*I*_h_ was elicited by a voltage protocol consisting of a family of hyperpolarizing steps to increasing negative potentials, from −40 to −150 mV from a holding potential of −20 mV, as previously described (Del Lungo et al., 2012).

### Solutions for electrophysiological recording

#### Extracellular solutions

Tyrode's solution (mM): D-(+)-glucose 10, NaCl 140, KCl 5.4, MgCl_2_ 1.2, CaCl_2_ 1.8, HEPES-NaOH 5.0, (pH 7.3); modified Tyrode's solution to measure *I*_h_ in HEK cells: Tyrode's solution with 25 mM KCl; modified Tyrode's solution to measure *I*_h_ in DRG neurons: Tyrode's solution with (mM): BaCl_2_ 2, MnCl_2_ 2, 4-aminopyridine 0.5, and KCl 25. *Intracellular solution* (mM): K-aspartate 130; Na_2_-ATP 5, MgCl_2_ 2, CaCl_2_ 5, EGTA 11, HEPES-KOH 10 (pH 7.2; pCa 7.0).

MEL55A (3-[(2Z)-4-{[(2R)-2-(3,4-dimethoxyphenyl)propyl] (methyl)amino}but-2-en-1-yl]-7,8-dimethoxy-2,3-dihydro-1H-3-benzazepin-2-one hydrochloride, see the formula in Supplemental Figure 1) was synthesized as reported previously (R5; Melchiorre et al., 2010). MEL55A and ivabradine solutions were obtained from stock solutions (10^−2^ M) in water and diluted in the different experimental solution to reach the desired final concentration.

### Data analysis and statistics

Current amplitude was obtained by fitting the time-dependent component of I_h_ current tracings from the peak initial current to the steady-state current with a mono- (in HEK) or bi-exponential function (in DGR neurons), which gave the best fitting results. In case of bi-exponential function, the time constant tau reported in Figures refers to the largest current component measured by the fitting. Current density was calculated as the difference between the peak current at the beginning of the hyperpolarizing step and the steady-state current, normalized to membrane capacitance. From the current-voltage relationship, specific current conductance was determined for each cell according to the equation:

$$\begin{array}{l} G_{HCN}={I\times \left(V_{m}-V_{rev}\right)}^{-1} \end{array}$$

where *G*_*HCN*_ is the conductance (pS/pF) calculated at membrane potential *V*_*m*_, *I* the current density (pA/pF), and *V*_*rev*_ (reversal potential) is calculated from the analysis of tail currents (Cerbai et al., 1994). The effect of forskolin on *I*_h_ activation, in the absence and presence of *I*_h_ blockade, was evaluated by tail current analysis, using a two-step protocol consisting of a first step varying from −50 to −130 mV, eliciting fractional *I*_h_, and a second step to −130 mV to activate the residual current. Activation curves of *I*_h_ were fitted with Boltzmann function

$$\begin{array}{l} G_{HCN\;}=gmax\times {\left\{1\;+exp\left[\left(V_{1/2}-V_{m}/k\right)\right]\right\}}^{-1} \end{array}$$

where V_½_ (mV) is the half-activation potential and *k* (mV) is the slope factor.

Concentration-effect curves were obtained at three different concentrations (1, 10, and 30 μM) and fitted to a Hill distribution

$$\begin{array}{l} y=E_{max}\times \left[x_{n}\times \left(k_{n}+x_{n}\right){\;}^{-1}\right] \end{array}$$

where *E*_*max*_ is the maximum effect, *k* corresponds to the concentration for half-maximal blocking effect (IC_50_), *x* is the drug concentration and *n* is the Hill coefficient.

Analysis of electrophysiological data and curve fitting was performed by using OriginPro 2015 (OriginLab Corporation, USA). Statistical comparison was performed with one-way ANOVA; the effect of compounds on *I*_h_ activation curve was evaluated by Multiple *t*-test (GraphPad PRISM v.5, USA). Behavioral measurements were performed on 12 mice for each treatment carried out in 2 different experimental sets. The analysis of variance of behavioral data was performed by one way ANOVA, a Bonferroni's significant difference procedure was used as *post-hoc* comparison. Data were analyzed using the “Origin 9” software (OriginLab, Northampton, USA).

All data are expressed as mean±SEM unless indicated. A probability value <0.05 was considered significant.

## Results

### Expression and localization of HCN isoforms in mouse DRG neurons

**Figure 1:**
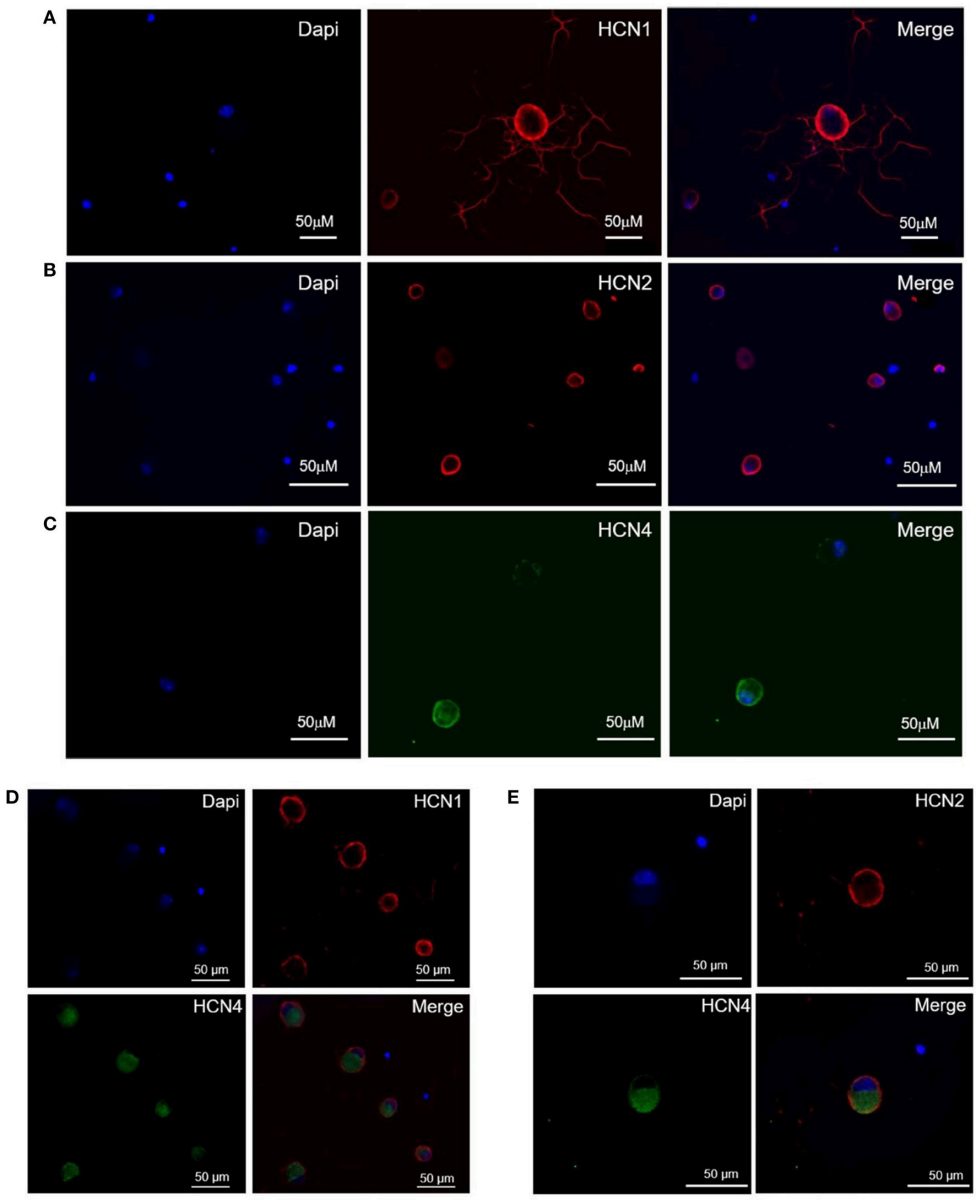
Expression and localization of HCN1 **(A)**, HCN2 **(B)**, and HCN4 **(C)** isoforms, and co-expression of HCN1/HCN4 **(D)** and HCN2/HCN4 **(E)** isoforms in DRG neurons. Immunofluorescence images of mouse DRG neurons show typical staining of HCN1 and HCN2 (red signal), HCN4 (green signal) and nuclei (blue signal). The white bar on each panel corresponds to 50 μM.

**Figure 2:**
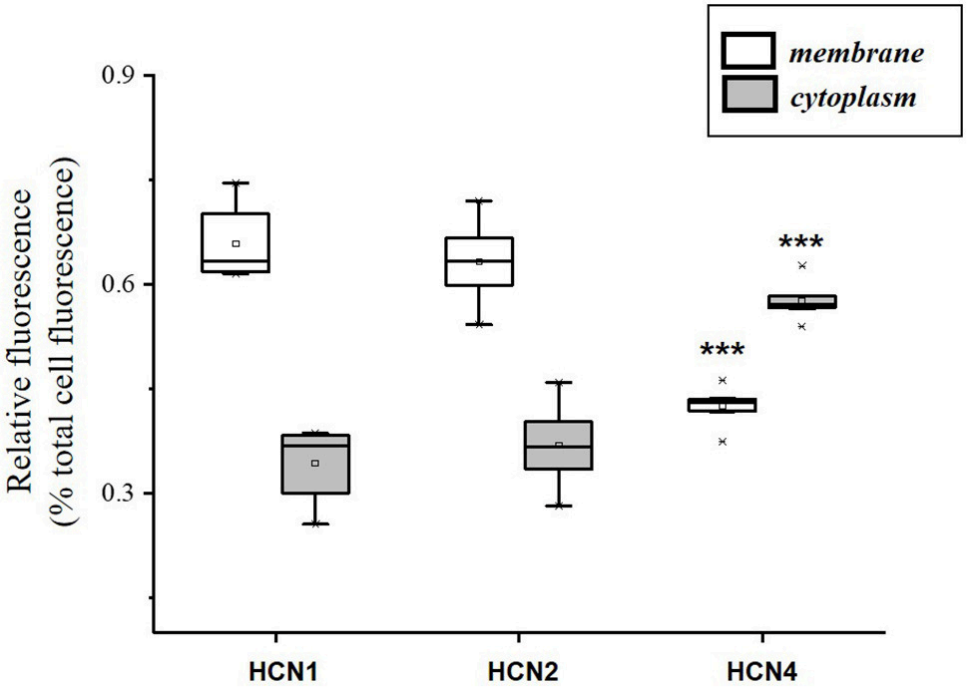
Semi-quantitative analysis of HCN isoforms expression in DRG neurons (*n* = 8) based on fluorescence. Each box represents the mean and range (25–75%) values measured by automated pixel analysis in the membrane (white boxes) or cytoplasmic area (gray boxes), normalized with respect to total cell fluorescence. ^***^p < 0.001 HCN4 vs. HCN1 and HCN2 (One-way ANOVA).

The population of DRG neurons in culture typically consists of cells with different dimensions for which, according to data in literature (Acosta et al., 2012; Schnorr et al., 2014), the proportion of HCN isoforms may vary depending on size. We observed that immunoreactivity for all three isoforms was present in DRG neurons (Figures 1A–C); however, their sublocalization was apparently different. The semiquantitative analysis reported in Figure 2 shows that HCN1 and HCN2 have a prevalent membrane localization; as for HCN4, immunoreactivity was detected at membrane as well as intracellularly, the proportion between the two compartments being significantly different from the other two isoforms. Co-localization experiments revealed a simultaneous expression of HCN1-HCN4 or HCN2-HCN4 in neurons, HCN1 and HCN2 isoforms showing prevalent membrane localization (Figures 1D,E).

### Isoform-selective effect of MEL55A in HEK293 expressing HCN

**Figure 3:**
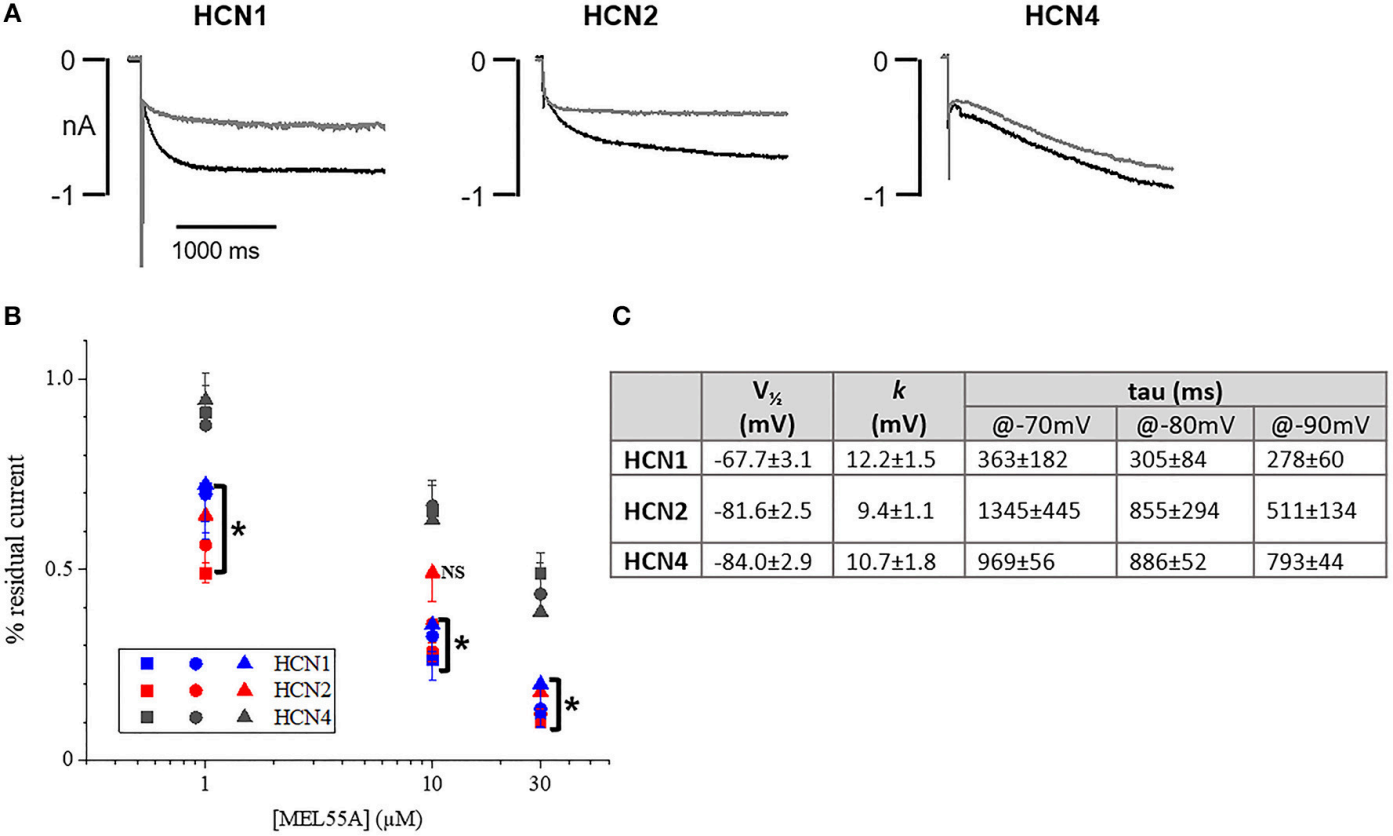
Effect of MEL55A on HCN isoforms heterologously expressed in HEK293 cells. **(A)** Representative tracings elicited by voltage steps to −80 mV, in the absence (black line) and presence (gray line) of 10 μM MEL55A. **(B)** concentration dependent blockade of HCN isoforms by 1, 10, and 30 μM measured at −70 mV (squares), −80 mV (circles), and −90 mV (triangles). Each point represents the mean ± S.E.M of 5–6 cells. ^*^p < 0.05 HCN1 or HCN2 vs. HCN4 by using One-way ANOVA Multiple comparison test. **(C)** Basic properties of activation curve for the three HCN isoforms heterologously expressed in HEK293 cells.

Based on expression profile and localization of HCN isoforms in DRG neurons and previously reported effect on maximal current in heterologously expressed channels (Melchiorre et al., 2010), we further assessed the properties of MEL55A (*R*-enantiomer, Supplemental Figure 1) in HEK293 cells at physiologically relevant potentials. Figure 3A shows the effect of 10 μM MEL55A on current evoked by hyperpolarizing step at −80 mV for the three isoforms, whose properties (*V*_½_ and time constant of activation, *tau*) are reported in Figure 3C. The percentage blockade of HCN current measured at −70, −80, and −90 mV with increasing concentrations of MEL55A (1, 10, and 30 μM) was significantly higher for HCN1 and HCN2 with respect to HCN4 at any concentration, with the exception of one point (HCN2 at −90 mV).

### *I*_*h*_ blockade by MEL55A in DRG neurons

**Figure 4:**
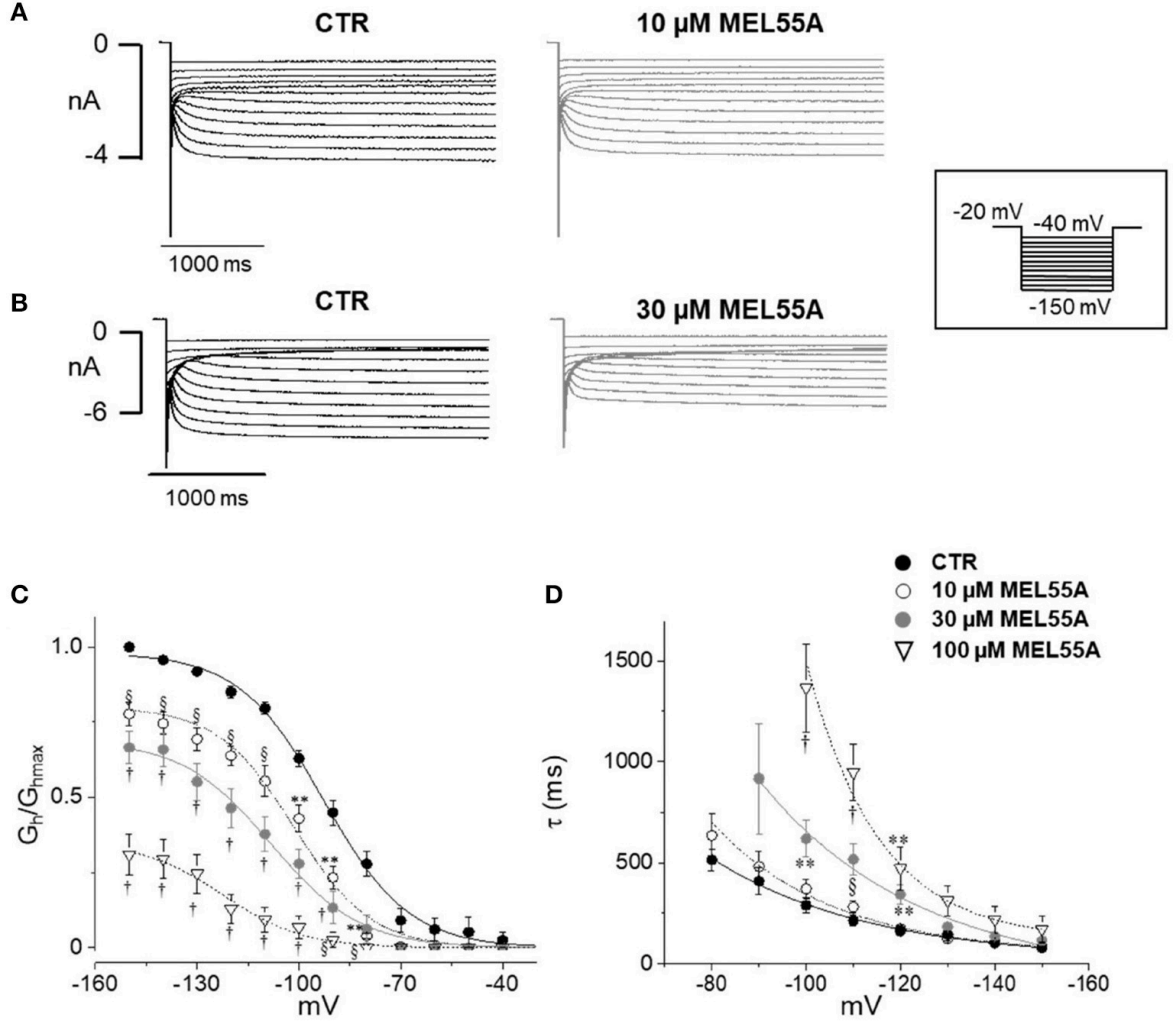
Effect of MEL 55A on *I*_h_ recorded from mouse DRG neurons. **(A,B)** Family of currents evoked by hyperpolarizing steps (voltage protocol in the inset), in the absence and presence of 10 **(A)** or 30 μM **(B)** MEL 55A. **(C,D)**. Average activation curves obtained w/wo MEL55A (10–100 μM) and corresponding time constant for current activation at different hyperpolarizing voltage steps. Each point represents the mean±S.E.M of 7–11 cells. ^*^p < 0.05 vs. CTR; ^**^p < 0.01 vs. CTR; § *p* < 0.001 vs. CTR; †*p* < 0.0001 vs. CTR by using Multiple *t* test.

Figure 4 shows typical current tracings evoked by hyperpolarizing steps in the absence and presence of 10 or 30 μM MEL55A. Average activation curve (Figure 4C) shows that MEL55A reduced *I*_h_ amplitude significantly (*p* < 0.0001, CTR vs all tested concentration) at any voltage step and in a concentration-dependent fashion. The effect was even more evident at less negative step potentials; at −80 mV (dashed line), MEL55A blocked about 80% of the available current, thus reducing *I*_h_ density from 0.28 ± 0.04 pS/pF in control (*n* = 11) to 0.06 ± 0.01 pS/pF (*n* = 7) at 10 μM and 0.06 ± 0.04 pS/pF (*n* = 7) at 30 μM. With 100 μM MEL55A (*n* = 7), no residual current was evoked by steps positive to −100 mV. At −120 mV, the percentage reduction of available current was 26% for 10 μM MEL55A, 46% for 30 μM, and 85% for 100 μM (*p* < 0.0001) (CTR: 0.85 ± 0.02 pS/pF, *n* = 11; 0.63 ± 0.03 pS/pF, *n* = 7; 0.46 ± 0.07 pS/pF, *n* = 7; 0.13 ± 0.05 pS/pF, *n* = 7, in the presence of 10, 30, and 100 μM MEL55A, respectively).

Due to a more pronounced effect at less negative potentials, the activation curve was apparently shifted to the left. The voltage of half-maximal activation (V_½_) was −92.7 ± 0.9 mV (*n* = 11) in control, −101.4±1.2 mV (*n* = 7, *p* < 0.0001), −108 ± 1.8 mV (*n* = 7, *p* < 0.0001), and −122 ± 2.7 mV (*n* = 7, *p* < 0.0001) in the presence of 10, 30, and 100 μM MEL 55A, respectively.

HCN isoforms have different kinetics and voltage-dependent properties, according to data obtained from homotetramer channels expressed in heterologous cells (Altomare et al., 2003; Stieber et al., 2004; Baruscotti et al., 2005). The time constant of activation of *I*_h_ in the absence and presence of MEL55A is shown in Figure 4D. The apparent shift of activation curve caused by MEL55A might be consistent with a more pronounced effect on HCN1 isoform, which activates at less negative potentials and exhibits a faster kinetics (see Figures 3A,C). In agreement with this hypothesis, current kinetics of activation was also slowed down by MEL55A (Figure 4D); of note, HCN1 and HCN2 also have a faster kinetics with respect to HCN4. At −120 mV, time constant of activation (τ) was 162 ± 22 ms in CTR, 173 ± 14 ms, 344 ± 50 ms (*p* < 0.01) and 473 ± 107 ms (*p* < 0.01) in the presence of 10, 30, and 100 μM MEL55A, respectively.

**Figure 5:**
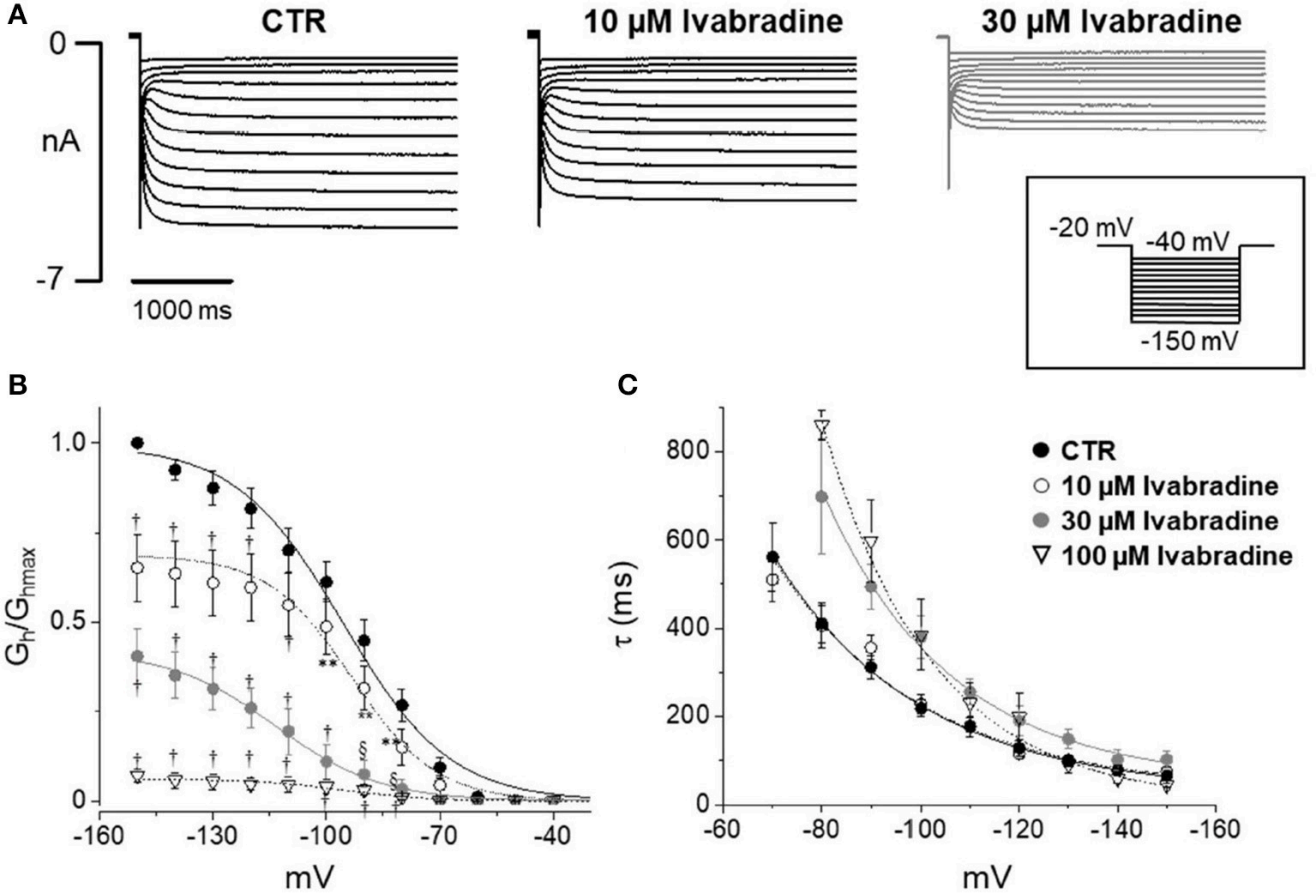
Effect of ivabradine on *I*_h_ recorded from mouse DRG neurons. **(A)** Typical current recordings in the absence or presence of 10 and 30 μM IVA; **(B,C)** Average activation curves and corresponding time constant for activation obtained w/wo ivabradine at different concentrations. Each point represents the mean±S.E.M of 9–12 cells. ^*^p < 0.05 vs. CTR; ^**^p < 0.01 vs. CTR; §*p* < 0.001 vs. CTR; †*p* < 0.0001 vs. CTR by using Multiple *t* test.

Figure 5 shows current tracings and activation curves obtained in the absence and presence of 10 and 30 μM ivabradine, a non-isoform-selective blocker of HCN. At the lowest concentration (10 μM, Figure 5C), ivabradine showed a similar qualitative effect but less marked current blockade (45% reduction at −80 mV) and V_½_ was unchanged (−95.5 ± 1.19 mV in CTR, *n* = 12 vs. −95 ± 1.3 mV with IVA, *n* = 9). The kinetics of current activation was not significantly affected (Figure 5D). Average membrane capacitance of tested neurons was 55 ± 9 pF, in line with data from cells cultured in similar conditions (Li and Baccei, 2014).

### Effect of *I*_*h*_ blockade on DRG membrane (voltage sag)

In DRG neurons, *I*_h_ likely plays a role in controlling membrane excitability (Tsantoulas et al., 2016; Sartiani et al., 2017); a characteristic of *I*_h_ activation is the occurrence of a “voltage sag” upon hyperpolarization, due to Na^+^ entry through HCN channels (Resta et al., 2016).

**Figure 6:**
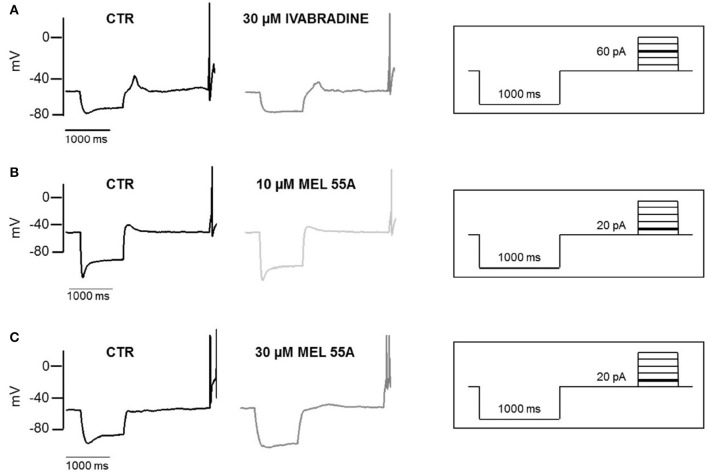
Effect of IVA and MEL55A on membrane potential and voltage sag in DRG neurons. Voltage sag and action potential evoked by a hyperpolarizing current (-100 pA) followed by a depolarizing pulse (60 pA) in control conditions and in the presence of 30 μM ivabradine **(A)** 10 μM MEL55A **(B)** and 30 μM MEL55A **(C)**. Current protocols are shown in inset on the right of corresponding traces.

Figure 6 shows examples of voltage sag generated by *I*_h_ in response to 1000 ms hyperpolarizing current steps (to −100 pA) in the absence and presence of MEL55A (10 and 30 μM) or ivabradine (30 μM). Consistent with *I*_h_ blockade at voltage steps around the “sag” membrane potential, current-clamp recordings showed a marked reduction of the amplitude of the voltage sag in the presence of MEL55A tested at 30 μM. We quantified contribution of HCN channel activity by measuring the percentage sag ratio, i.e., the difference between the peak voltage (V_peak_) and steady-state voltage (V_ss_), normalized to V_peak_. The voltage sag ratio decreased from 22.7 ± 3.6 to 14.0 ± 2.6% in the presence of 30 μM MEL55A (*n* = 12, *p* < 0.05). Similar effects were observed with ivabradine at the same concentration (26.4 ± 5.2% in CTR vs. 12.0 ± 2.1% with 30 μM IVA, *n* = 12).

### MEL55A counteracts the effect of cAMP on *I*_*h*_ activation

**Figure 7:**
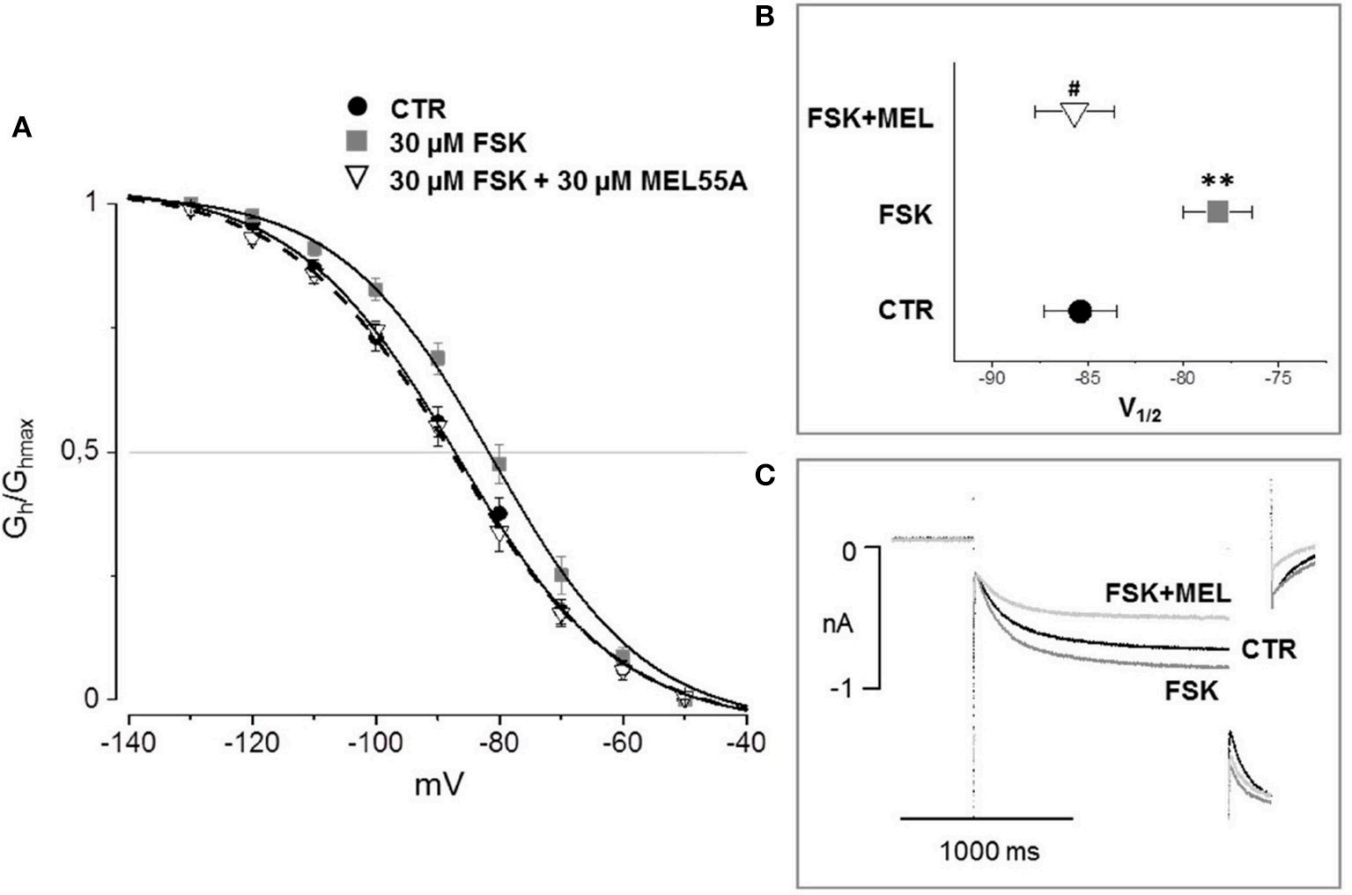
Effect of MEL 55A on *I*_h_ amplified by FSK in DRG neurons. Average activation curves **(A)** obtained in control (solid circles), with FSK (gray squares) and with FSK + MEL55A 30 μM (open triangles) and corresponding V_½_ **(B,C)**: typical current tracings measured during a double step protocol, evoked by hyperpolarization to −90 mV followed by a brief step to −130 mV. *N* = 11, ^**^p < 0.01 vs. CTR; #*p* < 0.01 vs. FSK.

**Figure 8:**
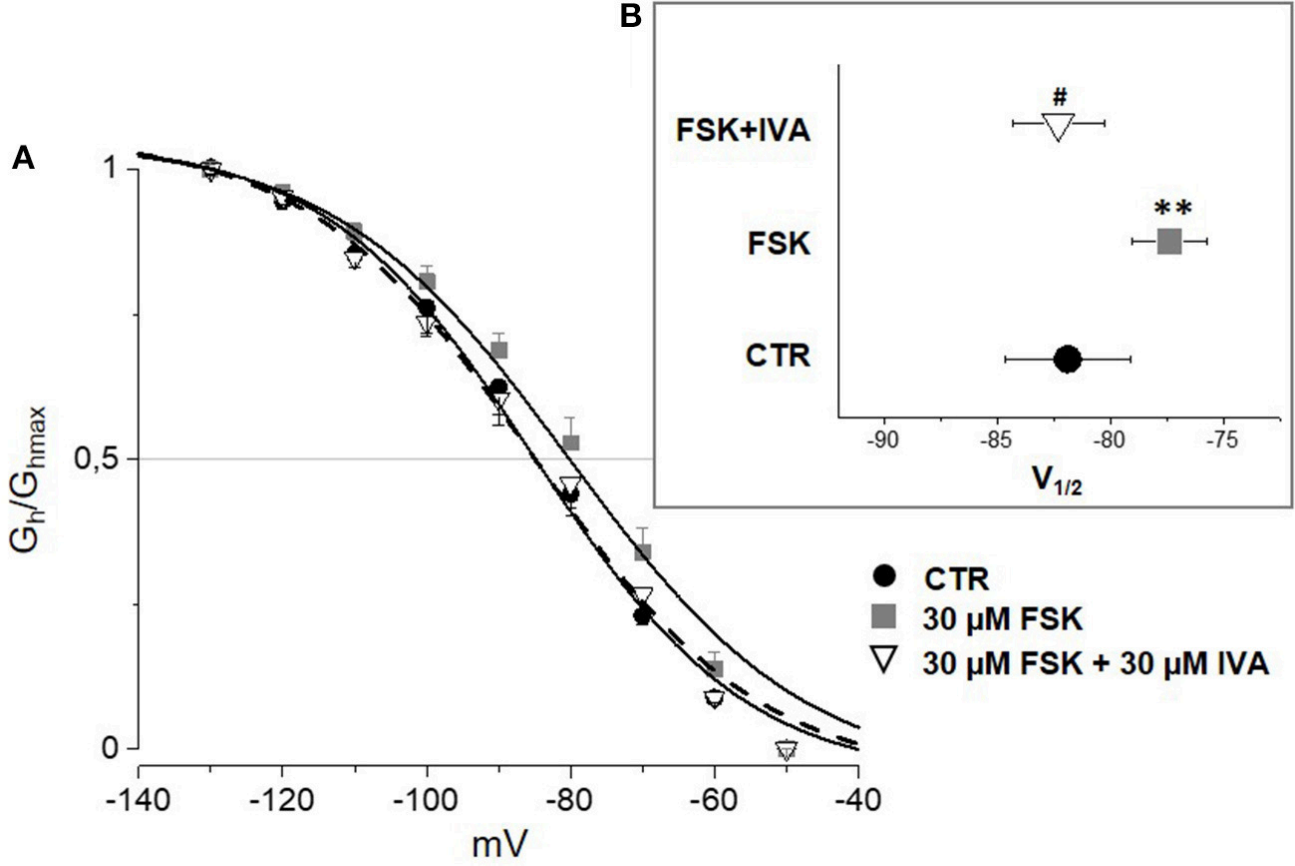
Effect of ivabradine (IVA) on *I*_h_ amplified by FSK in mouse DRG neurons. Average I–V curves **(A)** obtained in control (solid circles), with FSK (gray squares) and with FSK+ 30 μM ivabradine (open triangles) and corresponding V_½_ **(B)**. *N* = 10, ^**^p < 0.01 vs. CTR; #*p* < 0.01 vs. FSK.

According to the literature (see Sartiani et al., 2017 for a review) and previously published data from some of us (Resta et al., 2018), the contribution of *I*_h_ to DRG neuron excitability is amplified by pathological conditions able to modify channel expression or properties, e.g., by increasing intracellular cyclic AMP levels. When challenged with 30 μM forskolin (FSK), a direct activator of adenylate cyclase, the activation curve of *I*_h_ was shifted rightward, V_½_ being −85.4 ± 1.9 mV in CTR vs. −78.2 ± 1.8 mV with FSK (*n* = 11, *p* < 0.05), with no significant changes in maximum current (CTR 649 ± 143 pA; FSK 600 ± 139 pA) (Figures 7A,B). Likewise, this result suggests a relevant contribution of HCN2 isoform to *I*_h_ current recorded in our DGR neurons (Resta et al., 2016). In these conditions, as a proof of concept, we tested the effect of 30 μM MEL55A, i.e., a concentration able to block *I*_h_ almost completely at relevant potentials (Figure 5) and showing a moderate yet statistically significant selectivity toward HCN1/HCN2 isoforms compared to HCN4 (Figure 3B). At this concentration, MEL55A was able not only to reduce maximum *I*_h_ amplitude (407 ± 104 pA, p < 0.05 vs. CTR and FSK), but also to revert the positive shift of V_½_ (−85.7 ± 2.1 mV, *p* < 0.01 vs. FSK). Figure 7C reports typical current tracings measured during a double step protocol, evoked by hyperpolarization to −90 mV followed by a brief step to −130 mV. Similar results were observed with ivabradine (Figure 8).

### Effect of MEL55A on neuron discharge

**Figure 9:**
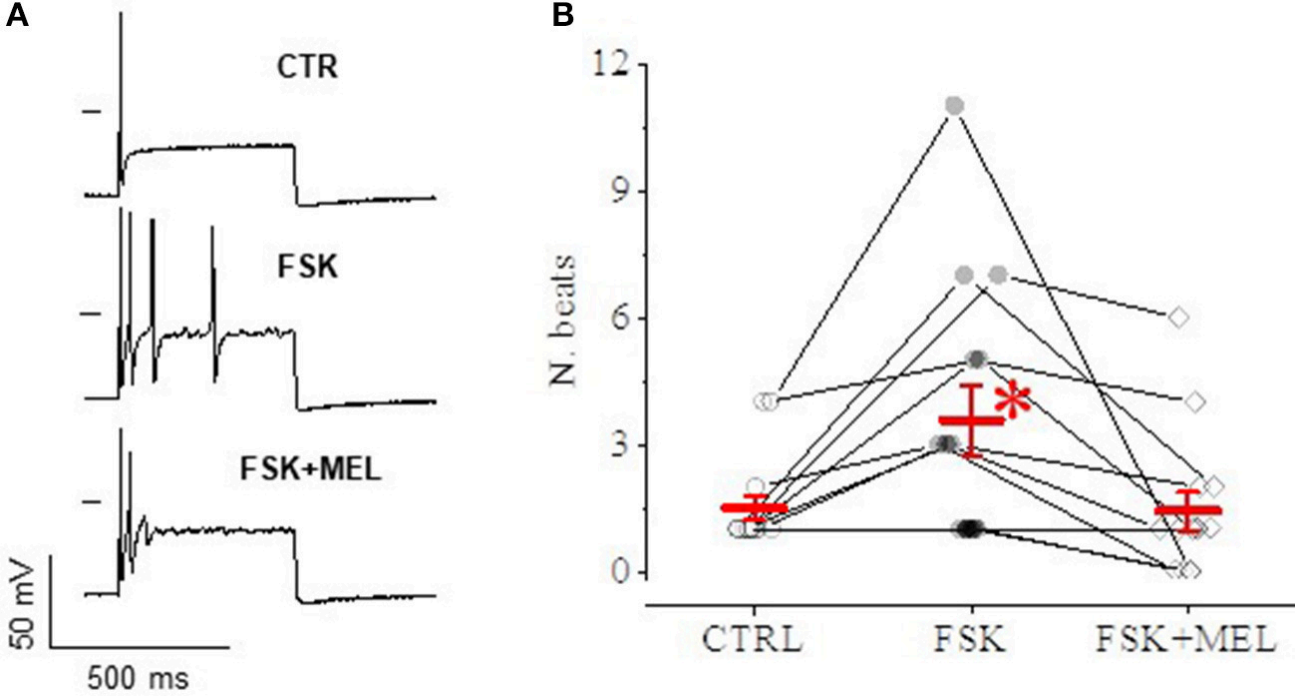
Effect of MEL55A on neuron discharge. An example is reported in panel A: a depolarizing step evokes a single AP in control condition **(A)**, followed by a series of APs after application of FSK (medium panel); 30 μM MEL55A on top of FSK reduces the number of APs (lower panel). **(B)** shows number of beats for individual cells challenged with such an experimental protocol (points) and corresponding mean ± S.E.M (in red) in *N* = 8 cells; ^*^p < 0.05 vs. CTR and FSK + MEL by using Multiple *t* test.

In current-clamp configuration, application of depolarizing steps evoked spontaneous action potential (AP), followed by quiescence (Figure 9A, top); superfusing with FSK—at the same concentration able to cause a rightward shift of *I*_h_ activation—led to the appearance of a series of spontaneous APs upon application of an identical depolarizing step (medium panel); 30 μM MEL55A on top of FSK reduced the number of APs (lower panel). The effect was consistently observed in 14 cells (Figure 9B).

### Effect of MEL55A on oxaliplatin-induced neuropathy

The ability of MEL55A to reduce hypersensitivity was tested in a mouse model of oxaliplatin-induced neuropathy. Two-weeks treatment with oxaliplatin progressively decreased the mice pain threshold as evaluated by the cold plate test. The licking latency decreased to 13.8 ± 1.1 s in comparison to vehicle-treated animals (23.2 ± 1.0 s, Table 1). The acute effect elicited by a single i.p. administration of MEL55A (30 mg kg^−1^) was evaluated on day 15. MEL55A induced a pain relief, lasting 45 min and peaking at 30 min, time at which the licking latency was restored close to control value. Under the same conditions, the effect of the same dose of ivabradine (30 mg kg i.p.) was shorter, disappearing after 30 min. In contrast, the compound EC18 (Del Lungo et al., 2012), previously characterized as HCN4-preferring compound, was inactive in this test (Table 1). All compounds did not modify the normal pain threshold of vehicle-treated animals (Table 1).

**Table 1:** Anti-allodynic effect of MEL55A and EC18 in comparison with ivabradine on oxaliplatin-induced neuropathy (Cold plate test).

	**Licking latency (s)**					
**Treatment**	**pretest**	**15 min**	**30 min**	**45 min**	**60 min**	**75 min**
vehicle + vehicle	23.2 ± 1.0	22.5 ± 0.6	22.8 ± 0.9	23.2 ± 0.4	21.9 ± 1.1	22.8 ± 1.5
vehicle + MEL55A 30 mg/kg	22.8 ± 1.9	24.7 ± 0.8	23.5 ± 1.3	22.8 ± 0.8	24.1 ± 0.8
vehicle + EC18 30 mg/kg	21.6 ± 1.2	23.7 ± 1.3	24.5 ± 0.8	22.1 ± 0.6	20.1 ± 0.9
vehicle + IVABRADINE 10 mg/kg	22.65 ± 1.3	20.4 ± 1.5	22.6 ± 1.1	23.7 ± 1.3	22.4 ± 0.9
oxaliplatin + vehicle	13.8 ± 1.1	12.6 ± 0.7	14.3 ± 0.5	13.9 ± 0.8	13.7 ± 0-7	12.5 ± 0.8
oxaliplatin + MEL55A 30 mg/kg	14.5 ± 0.6	19.4 ± 0.8^*^	21.9 ± 0.9^*^	18.5 ± 0.8^*^	15.5 ± 0.9	12.6 ± 0.6
oxaliplatin + EC18 30 mg/kg	13.6 ± 1.0	12.2 ± 0.9	13.0 ± 0.7	14.2 ± 0.5	13.2 ± 1.2
oxaliplatin + IVABRADINE 30 mg/kg	13.8 ± 1.4	20.7 ± 1.5^*^	19.2 ± 1.1^*^	17.4 ± 0.9	15.6 ± 1.3

Oxaliplatin (2.4 mg/kg) was dissolved in 5% glucose solution and administered i.p. on days 1–2, 5–9, 12–14. On day 15, compounds (30 mg/kg, dissolved in saline containing 5% DMSO) were i.p. administered. Pain-related behavior (i.e., lifting and licking of the hind paw) was observed by the cold plate test and the time (s) of the first sign was recorded.

* *P < 0.05 in respect to the oxaliplatin treated mice. Each value represents the mean of 12 mice performed in 2 different experimental sets*.

## Discussion

Our results demonstrate that MEL55A, exhibiting preferential blockade of heterologously expressed HCN2 and HCN1 isoforms, could diminish the amplitude of *I*_h_, either in basic conditions and after stimulation by intracellular cAMP, and reduce cell excitability in mouse DRG neurons in culture. To our knowledge, this is the first demonstration that preferential blockade of HCN2 and HCN1 channels, over HCN4 isoform, was able to modulate the electrophysiological properties of DRG neurons similarly to that reported for classical *I*_h_ blockers, ZD7288 and ivabradine (Chaplan et al., 2003; Descoeur et al., 2011).

MEL55A is an analog of zatebradine characterized by reduced-flexibility and a stereogenic center (R configuration) close to the dimethoxyphenyl ring whose synthesis and preliminary screening in HEK cells has been previously described (R5 in Melchiorre et al., 2010). A preferential HCN1/HCN2 blockade was observed on heterologously re-expressed, fully activated current (-120 mV) and further suggested by stereoselectivity: in fact, the blocking potency of the S-enantiomer on maximally activated *I*_h_ (i.e., at −120 mV) was quantitatively smaller and similar for the three HCN isoforms. Thus, we extended previous results and showed that MEL55A preferentially blocked HCN1/HCN2 isoforms over HCN4 also when tested on heterologously re-expressed current evoked by steps at physiologically relevant potentials (−70 to −90 mV). This observation prompted us to naïve cells where these isoforms may play a relevant physiological role in controlling excitability, i.e., DRG neurons (Tibbs et al., 2016).

According to our hypothesis, MEL55A reduced *I*_h_ amplitude in DRG neurons; concurrently, when tested at 30 μM, it halved the amplitude of voltage sag upon hyperpolarization and inhibited neuron excitability following depolarizing steps. In line with published data (Young et al., 2014), the effect on *I*_h_ was similar to that observed with ivabradine.

Translating results on HCN isoforms expressed in recombinant systems to naïve cells is always difficult for several reasons. First, h-channels in DRG neurons are likely heterotetramers, although the exact stoichiometry is uncertain, while only homotetramers are expressed in our HEK cells. Second, the functional and pharmacological properties of naïve *I*_h_ also depends on post-translational channel modification including membrane translocation, presence of beta subunits (e.g., MiRP1) and co-localization with caveolin-3, which modify channel properties, in particular voltage-dependence and current amplitude (see Sartiani et al., 2017 for a comprehensive review).

In our cultured DGR neurons, also in agreement with data in literature (Acosta et al., 2012), we observed a well-defined expression and membrane localization for HCN1 and HCN2 isoforms by immunostaining and semi-quantitative analysis, while HCN4 staining was mainly detected at cytoplasmic level. As for electrophysiological properties, time constant (*tau*) for *I*_h_ activation measured in DRG neurons at −100 mV was around 250 ms, similar to values reported in previous studies in the same cells (Gao et al., 2012). Interestingly, *tau* value lays midway those measured for heterologously expressed HCN1 and HCN2 isoforms in our experimental conditions (at −100 mV: 182 ± 29 and 312 ± 59 ms, respectively; *tau* for HCN4: 690 ± 39 ms). Overall, the electrophysiological properties of *I*_h_ measured in our experimental conditions are consistent with HCN1/HCN2 characteristics in recombinant systems. Although the functional presence of HCN4 in naïve h-channels cannot be ruled out completely and a detailed characterization of HCN isoform contribution to *I*_h_ was beyond the scope of this study, it is worth to recall that the selective HCN4 blocker, EC18, was completely ineffective on *I*_h_ measured in DRG neurons in the same conditions up to a concentration of 100 μM (Del Lungo et al., 2012).

The “apparent” negative shift of activation curve caused by MEL55A observed for *I*_h_ in DRG neurons is not completely surprising, if one recalls the so-called “current dependence” of HCN blockade originally reported for ivabradine (Bucchi et al., 2002). Briefly, blockade by ivabradine is removed when current flows inwardly through the open f-channels, while develops rapidly when channels deactivate at depolarized voltages. Channel unblock during persistent opening (i.e., hyperpolarization) has also been observed (Bucchi et al., 2006). Finally, HCN1 blockade by ivabradine occurs also, at least in part, when the channel is closed. If MEL55A behaves similarly, percentage blockade of HCN1 and HCN2 might be favored at less negative potentials, due to the combination of (i) preferential selectivity for these isoforms, (ii) blockade (also) of closed channels, and (iii) weak washout of the drug by current flowing through the pore. Instead, the blockade could be partially removed at more negative potentials, when large inward flow of Na^+^ ions (and K^+^, depending on voltage) favors unblock of the channel. Due to its possible (patho)physiological implications for MEL55A or other compounds, this hypothesis deserves to be proved by appropriate testing in future experiments.

The possibility to modulate DRG excitability by *I*_h_ blockade is not new and it has been proved in previous studies *in-vivo* and *in-vitro*. Administration of ZD7288 reduced *I*_h_ by 80%; such an effect was accompanied by the suppression of the voltage sag, i.e., a time dependent depolarization consequent to injection of hyperpolarizing current due to Na^+^ entry through HCN channels (Gao et al., 2012). A similar effect was achieved with ivabradine (Noh et al., 2014; Young et al., 2014), the only *I*_h_ blocker, specific bradycardic agent commercially available in angina and heart failure (Camici et al., 2016; Ponikowski et al., 2016; Psotka and Teerlink, 2016). Unfortunately, the exploitation of ivabradine as analgesic drug is precluded justly by its *side* (i.e., bradycardic) effect, a consequence of the lack of drug's selectivity toward HCN isoforms (or slight HCN4 selectivity, Bucchi et al., 2006) In this context, the possibility to exploit differences in HCN isoform expression among tissues seems attractive: indeed, HCN4, the most contributor for sinoatrial pacemaking (Moosmang et al., 2001; Stieber et al., 2004; Baruscotti et al., 2011), largely overexpressed in diseased ventricular tissue (Stillitano et al., 2008; Suffredini et al., 2012) seems to play a negligible role in neuronal excitability.

Several studies support the expression and role of HCN1 and HCN2 isoforms in DRG neurons in physiological conditions; nevertheless, the differential role of HCN subtypes in modulating pain is still a matter of debate (Acosta et al., 2012; Schnorr et al., 2014; DiFrancesco and DiFrancesco, 2015). The contribution of the HCN2 isoform in inflammatory pain is suggested by the involvement of cAMP-mediated pathways (e.g., by prostaglandin E2 receptor stimulation) and the efficacy of HCN2 silencing in relieving neuropathic pain (Emery et al., 2011, 2012; Resta et al., 2016). In our DRG neurons, forskolin, an adenylate cyclase activator, shifted current activation toward less negative potentials by 8–10 mV. Hence, the capability of MEL55A to reduce *I*_h_ amplitude and counteract the effect of forskolin on current activation is particularly relevant, suggesting that homomeric or heteromeric channels containing HCN2 subunits may represent targets of MEL55A blockade in hyperalgesia.

At the same time, several conditions such as nerve injury, antineoplastic agents and diabetes appear to increase HCN1 expression and function (Jiang et al., 2008; Tu et al., 2010; Descoeur et al., 2011). Different results have been also obtained depending on stress condition (acute vs. chronic pain) or stimulus (heat vs. mechanic hypersensitivity) (Schnorr et al., 2014). As a matter of fact, the relative expression of HCN1 and HCN2 isoforms is likely variable among neuron subsets, depending on size, location, and soundly reflecting different functions (Tibbs et al., 2016). Thus, a pharmacological tool aimed to target both HCN1 and HCN2 isoforms, but less potent on HCN4, might represent a suitable strategy in different settings.

To see if the *I*_h_-blocking properties of MEL55A observed *in vitro* could translate into a pharmacological effect *in vivo*, we tested the antihyperalgesic properties of this compound in a mouse model of oxaliplatin-induced neuropathy. Indeed, a rigorous pharmacological approach requires a previous pharmacokinetic assessment, which is not available at present and will be object of further studies. However, 30 mg/Kg MEL55A clearly showed a pain-relieving effect, which was longer-lasting when compared to the same dose of ivabradine. The significant antineuropathic effect of MEL55A could be explained, similarly to what observed with MEL57A, by *I*_h_ gain-of-function caused by oxaliplatin treatment (Resta et al., 2018), possibly related to overexpression of the ancillary subunit MiRP-1. When co-expressed in heterologous systems, this subunit is known to modulate both HCN1 and HCN2 isoforms by accelerating their kinetics (Yu et al., 2001; Qu et al., 2005). It is worth noting that, in the same conditions, the HCN4-preferring agent EC18 (Del Lungo et al., 2012) lacked anti-allodynic effect (Table 1), further suggesting that HCN4 plays a minor role in neuropathic pain (Emery et al., 2011; Resta et al., 2018).

## Limitations and conclusions

At present, our results do not allow inferring that MEL55A represents a *drug candidate*; this aim was beyond the scope of this study. We must acknowledge several limitations, requiring further test: the lack of extensive pharmacodynamic (e.g., effect on channels different from HCN) and pharmacokinetics data and high concentrations used for our preliminary *in-vivo* proof of concept. However, present results are promising in view of drug design aimed to develop novel antinociceptive strategies and original tools able to discriminate the contribution of HCN isoforms in different pathophysiological conditions, which remains a challenging issue. The shortage of pharmacological agents able to treat neuropathic pain, a quite prevalent form of chronic pain, represents an increasingly medical burden (Finnerup et al., 2015). The availability of small molecules with selectivity toward HCN2 channels might represent a safe and effective strategy (Tsantoulas et al., 2016). In line with our recent findings (Resta et al., 2018), present data further support that, from a pharmacological point of view, this approach is affordable and deserves to be further exploited in more integrated models.

## Author contributions

EC, MR, MD, FR, and RC: conception and design of experiments. LD, MD, and FR: collection, analysis, and interpretation of patch-clamp data. LDCM and CG: collection, analysis, and interpretation of behavioral data. VS, MD, and AL: design and analysis of molecular biology and immunohistochemistry. MM and MR: drug design and synthesis. LS, GM, MR, and EC: drafting the article or revising it critically for important intellectual content. All authors approved the final version of the manuscript.

### Conflict of interest statement

The authors declare that the research was conducted in the absence of any commercial or financial relationships that could be construed as a potential conflict of interest.

## Abbreviations

AP: action potential
DRG: dorsal root ganglion
HCN: hyperpolarization activated cyclic nucleotide-gated channels
HEK293: human embryonic kidney cells
*I*_h_: hyperpolarization-activated current
FSK: forskolin
V_½_: voltage of half-maximal current activation
