# Supplementary figures and images for: Selective Blockade of HCN1/HCN2 Channels as a Potential Pharmacological Strategy Against Pain
Source: Front Pharmacol. 2018 Nov 8;9:1252. doi: 10.3389/fphar.2018.01252 (PMC6237106; doi:10.3389/fphar.2018.01252)

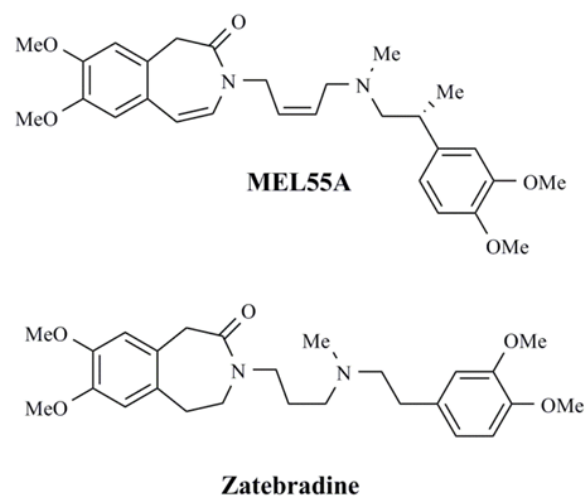

**Supplemental Figure 1.** Chemical structure of MEL55A and zatebradine as free bases.

Supplement: Supplementary file 1 [file Image_1.pdf]
